# Copy Number Variation Pattern for Discriminating MACROD2 States of Colorectal Cancer Subtypes

**DOI:** 10.3389/fbioe.2019.00407

**Published:** 2019-12-19

**Authors:** ShiQi Zhang, XiaoYong Pan, Tao Zeng, Wei Guo, Zijun Gan, Yu-Hang Zhang, Lei Chen, YunHua Zhang, Tao Huang, Yu-Dong Cai

**Affiliations:** ^1^School of Life Sciences, Shanghai University, Shanghai, China; ^2^Department of Biostatistics, University of Copenhagen, Copenhagen, Denmark; ^3^Key Laboratory of System Control and Information Processing, Institute of Image Processing and Pattern Recognition, Ministry of Education of China, Shanghai Jiao Tong University, Shanghai, China; ^4^Key Laboratory of Systems Biology, Institute of Biochemistry and Cell Biology, Chinese Academy of Sciences, Shanghai, China; ^5^Institute of Health Sciences, Chinese Academy of Sciences, Shanghai Jiao Tong University School of Medicine and Shanghai Institutes for Biological Sciences, Shanghai, China; ^6^Shanghai Institute of Nutrition and Health, Shanghai Institutes for Biological Sciences, Chinese Academy of Sciences, Shanghai, China; ^7^College of Information Engineering, Shanghai Maritime University, Shanghai, China; ^8^Shanghai Key Laboratory of PMMP, East China Normal University, Shanghai, China; ^9^Anhui Province Key Laboratory of Farmland Ecological Conservation and Pollution Prevention, School of Resources and Environment, Anhui Agricultural University, Hefei, China

**Keywords:** copy number variation, MACROD2, colorectal cancer, subtype, classification

## Abstract

Copy number variation (CNV) is a common structural variation pattern of DNA, and it features a higher mutation rate than single-nucleotide polymorphisms (SNPs) and affects a larger fragment of genomes. CNV is related with the genesis of complex diseases and can thus be used as a strategy to identify novel cancer-predisposing markers or mechanisms. In particular, the frequent deletions of mono-ADP-ribosylhydrolase 2 (*MACROD2*) locus in human colorectal cancer (CRC) alters DNA repair and the sensitivity to DNA damage and results in chromosomal instability. The relationship between CNV and cancer has not been explained. In this study, on the basis of the genome variation profiling by the SNP array from 651 CRC primary tumors, we computationally analyzed the CNV data to select crucial SNP sites with the most relevance to three different states of *MACROD2* (heterozygous deletion, homozygous deletion, and normal state), suggesting that these CNVs may play functional roles in CRC tumorigenesis. Our study can shed new insights into the genesis of cancer based on CNV, providing reference for clinical diagnosis, and treatment prognosis of CRC.

## Introduction

Copy number variation (CNV) is a common structural variation pattern of DNA; it is defined as a >1 kb genomic segment with a different copy number compared with the reference genome, leading to gains, or losses of multiple DNA sites that are either microscopic or submicroscopic (Redon et al., 2006). CNV features a higher mutation rate than single-nucleotide polymorphisms (SNPs) and affects a larger fragment of genomes (Zhang et al., 2009). For a large number of CNVs generated in the human genome, one of the known mechanisms is DNA recombination, which includes non-allelic homologous recombination and non-homologous end-joining. Recently, a new mechanism based on DNA error replication has been discovered. Named the “Fork stalling and switching” model, this mechanism can explain complex-structure CNVs that do not conform to non-allelic homologous recombination or non-homologous end-joining.

With the development of high-resolution SNP arrays, identifying large-scale CNVs in thousands of samples has been possible (Beroukhim et al., 2010). Studies have demonstrated that CNV is related to the genesis of Mendelian diseases, sporadic diseases, and susceptibility to complex diseases (Yang et al., 2008; De Cid et al., 2009; Willer et al., 2009; Sato et al., 2014; Zhang et al., 2014). CNVs also play a potential role in cancer risk, and the genome-wide copy number analysis can be used as a strategy to identify novel cancer-predisposing markers or mechanisms (Kuiper et al., 2010). Ding et al. (2010) reported that the genome of primary tumors is diverse and frequently includes gene rearrangements and copy number variations. Shlien et al. (2008) used high-density oligonucleotide arrays to compare the genomes of healthy population and a Li–Fraumeni cancer predisposition disorder (LFS) cohort and observed that CNV in the cell adhesion gene mixed-lineage leukemia translocated 4 (*MLLT4*) is associated with LFS, in which patients always harbor a germline heterozygous mutation of the tumor suppressor gene *TP53* and experience a high probability of developing early-stage breast, sarcoma, brain, and other tumors. Scrima et al. (2012) revealed that 24, 31, and 26% of patients with lung adenocarcinoma achieved a copy number gain in adenylate kinase (*AK*) 1, *AK2*, and phosphoinositide-3-kinase, catalytic, alpha polypeptide (*PI3KCA*), respectively, via fluorescence *in situ* hybridization.

Evidence has recognized CNV as one of the most important genomic alterations affecting cancer pathogenesis (Hermsen et al., 2002), whereas chromosomal instability and allelic imbalance at certain chromosomal loci play crucial roles in most sporadic cases of colorectal cancer (CRC) (Zanke et al., 2007). CRC is the fourth most common cancer and the second leading cause of cancer death worldwide, with over 1.1 million new cancer cases and 880,000 deaths estimated in 2018 (Bray et al., 2018). For better assessment of the progression of CRC, the Dukes staging system was proposed as a common classification system for CRC (Dukes, 1932). Four stages of CRC are defined by such system depended on the degree of colorectal pathology. Dukes A represents the invasion of tumor cells into but not through the bowel wall. Patients in Dukes A stage usually have better outcomes with over 90% 5-year survival. When tumor grows through the muscle layer of the bowel but not infiltrate into lymph nodes, it will be identified as Dukes B stage. Dukes C refers to the spread of cancer to at least one lymph node close to the bowel. And lastly, widespread metastases of tumor cells in CRC, also called advanced CRC, indicate the stage of Dukes D. The clear stage of CRC contributes to the decision making in clinical treatment, and also provides a detailed description for the pathology research.

Frequent deletions of the mono-ADP-ribosylhydrolase 2 *(MACROD2*) locus in human CRC alter DNA repair and sensitivity to DNA damage and result in chromosomal instability (Sakthianandeswaren et al., 2018). In addition, *MACROD2* deletion in CRC is significantly associated with the extent of malignancy, indicating that *MACROD2* acts as a haploin-sufficient tumor suppressor, with the loss of function promoting chromosome instability and thereby driving cancer evolution.

In this study, based on the genomic variation profiling by SNP array from 651 CRC primary tumors (Sakthianandeswaren et al., 2018), the log R ratio (LRR) and B allele frequency data (BAF) of each SNP site were exported using two types of hybridization probes specific to two types of known alleles (Wang et al., 2007), and the SNP genotype also can be determined by the ratios of the hybridization intensities of two types of probes. The genotype of SNPs located in the region of *MACROD2* was used to represent the genotype state of *MACROD2*, which means that the individuals with the loss of both alleles in at least one SNP site in *MACROD2* will be classified into the state of homozygous deletion, and the deletion of only one allele indicates the heterozygous deletion status. A wild-type stage or normal stage refers to no deletion happened in *MACROD2*. Following that, each patient was classified into one of the three states: heterozygous deletion, homozygous deletion, and normal state in our study. We computationally analyzed the CNV data to select the crucial SNP sites showing the most relevance to the four Dukes stages of CRC (A, B, C, and D) and three different states of *MACROD2* (heterozygous deletion, homozygous deletion, and normal state), suggesting that these CNVs may play functional roles in CRC tumorigenesis. We constructed a classifier with high accuracy to group individuals into the corresponding state categories. This classification model also provides a meaningful list of genomic loci that perform important functions in the development and progression of cancers. To date, the relationship between CNV and cancer has not been exactly explained. Our study can shed new light on the genesis of cancer based on CNV, providing reference for the clinical diagnosis and treatment prognosis of CRC.

## Materials and Methods

In this study, we first used the minimum redundancy and maximum relevance (mRMR) method (Peng et al., 2005) to analyze all features. Irrelevant features were discarded and the rest features were ranked in a feature list, which was further fed into the incremental feature selection (IFS) (Liu and Setiono, 1998) to obtain the optimum features and extract the classification rules for readable explanation. We adopted the same computational pipeline to separately analyze four kinds of carefully organized datasets, including the CRC stage with LRR or BAF and the *MACROD2* status with LRR or BAF.

### Datasets

The LRR and BAF data on 651 CRC primary tumors obtained using the Illumina Human610-Quad v1.0 BeadChip were downloaded from Gene Expression Omnibus under the accession number GSE115145 (Sakthianandeswaren et al., 2018). The LRR and BAF were calculated with GenomeStudio (Illumina). The 651 CRC samples can be divided into four stages: 60 stage A samples, 208 stage B samples, 297 stage C samples, and 86 stage D samples. Based on *MACROD2* status, 441 wild-type samples, 137 heterozygous deletion samples, and 73 homozygous deletion samples were obtained. Each sample was represented by 620,901 SNP features.

### Feature Selection

As mentioned above, each sample was represented by lots of SNP features. Clearly, not all of them were highly related to classification of these samples. Thus, we employed some powerful feature selection methods to analyze all features. The analysis procedures included three stages. The first stage was to exclude irrelevant features; the second one was to sort rest features; the last stage was to construct optimal classifier with optimum features and classification rules with the help of IFS method, support vector machine (SVM) (Corinna Cortes, 1995), and repeated incremental pruning to produce error reduction (RIPPER) (Cohen, 1995).

The purpose of the first stage was to exclude irrelevant features. To this end, all features were evaluated by the mRMR method. The mRMR method was a mutual information (MI)-based feature selection method (Peng et al., 2005; Li et al., 2019). The importance of each feature was evaluated by its MI to class labels. It is clear that the higher the MI values were, the more important the features were. After a threshold for MI value was set, irrelevant features can be excluded.

After irrelevant features were excluded, rest features were assessed by mRMR method in another way in the second stage. In detail, rest features were ranked in a feature list in terms of their relevance to class labels and redundancies to other features. The feature subset consisting of some top features in the list can be deemed to be the optimal feature combination with highest relevance to class labels and lowest redundancies among these features, which can provide a powerful discrimination. In this study, we used the mRMR program downloaded from http://home.penglab.com/proj/mRMR/index.htm. Default parameters were adopted.

In the third stage, we ran a two-stage IFS with a classification algorithm to select the optimum features for building the optimal classifier or construct classification rules. In the first stage, a series of feature subsets with a step 10 was generated, where feature subset 1 consists of the top 10 features, feature subset 2 consists of the top 20 features, and so on. Then, for each feature subset, a classifier was trained on the samples consisting of the features from the feature subset, and this classifier was evaluated using 10-fold cross-validation (Kohavi, 1995). An interval [min, max] with a good performance was determined. In the second stage, a series of feature subsets within the interval [min, max] was generated to further select the final optimum features or construct classification rules. Based on these optimum features, an optimal classifier can be built.

### SVM

SVM attempts to identify a hyper plane with a maximum margin between two groups of samples, and it has been widely used in biological data studies (Pan and Shen, 2009; Mirza et al., 2015; Cai et al., 2018; Chen et al., 2018, 2019; Zhou et al., 2019). In this work, we used a multi-class SVM with a one vs. rest strategy. The multi-class SVM consists of multiple binary SVMs, and each SVM classifies the samples of one class from the rest of the classes. When predicting the class for a new sample, the SVM predicts the sample's label corresponding to the class with the highest probability. This study adopted the SVM implemented by a tool “SMO” in Weka.

### Rule Learning

To understand how a classification model makes a prediction, we used rule learning to extract the readable classification rules. A rule consists of an IF-THEN relationship between features and output labels, such as IF SNP1 <= 0.7 AND SNP2 >= 1.02; THEN stage = “A.” In this study, we applied RIPPER (Cohen, 1995), which is implemented by a tool “JRip” in Weka. RIPPER consists of two stages, including the rule building stage and rule optimization stage.

### SMOTE

As mentioned in the *Datasets* section, 651 CRC samples were classified into three or four classes. The sizes of classes varied a lot. Thus, investigated datasets were imbalanced. For this type of dataset, the performance of an ordinary classifier is dependent on the biggest class. To tackle this problem, Synthetic Minority Over-sampling Technique (SMOTE) (Chawla et al., 2002; Wang et al., 2018; Zhang et al., 2019) was employed in this study, which is a oversampling method. This method can produce some new samples and pour into minority class, thereby making all classes having equal sizes. In this study, for the BAF/LRR dataset of CRC stage, new samples were generated by SMOTE for classes of stages A, B, and D, while new samples were yielded by SMOTE for classes of heterozygous deletion and homozygous deletion for BAF/LRR dataset of *MACROD2* status.

In this study, we adopted the SMOTE program implemented by python, which was downloaded at https://github.com/scikit-learn-contrib/imbalanced-learn.

## Results

**Figure 1:**
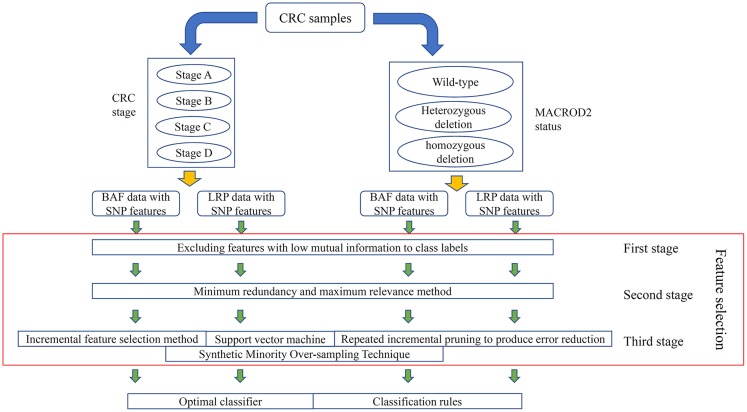
Entire procedures to analyze the log R ratio (LRR) and B allele frequency (BAF) data on colorectal cancer (CRC) primary tumor samples. CRC samples are classified into four stages; at the same time, they can also be classified into three classes according to their MACROD2 status. For each classification, two datasets with LRR and BAF, respectively, were constructed. Four datasets were obtained in total, in which single-nucleotide polymorphism (SNP) features were used to represent each CRC sample. A feature selection procedure, including three stages, was adopted to analyze all SNP features. Finally, an optimal classifier and several classification rules were accessed for each dataset.

In this study, we separately analyzed the four kinds of carefully organized datasets with a three-stage feature selection method. Whole procedures are illustrated in Figure 1.

For the first stage, we set the threshold of MI values to be 0.01; i.e., features receiving the MI values larger than 0.01 were kept. The number of remaining features for BAF/LRR dataset of CRC stage was 47515/44931, while it was 20839/20973 for BAF/LRR dataset of *MACROD2* status. Then, in the second stage, remaining features in each dataset were ranked by the mRMR method. Obtained feature lists are provided in Tables S1–S4. The third stage employed the IFS method and classification algorithms to extract optimum features and construct classification rules. The key results are provided in Tables 1–**4**.

**Table 1:** Performance of classification models on BAF dataset of CRC stage with IFS method.

**Classifier**	1st-stage IFS^*^		2nd-stage IFS^*^	**Number of rules**
	**Highest point**	**Turning point**		
SVM	0.9653 (35,440)	0.9007 (8,790)	0.9008 (8,797)	—
RIPPER	0.2932 (8,500)	0.2692 (2,170)	0.2745 (2,075)	30

* *These performances are measured by MCC; numbers of used features are listed in brackets*. *BAF, B allele frequency; CRC, colorectal cancer; IFS, incremental feature selection; SVM, support vector machine; RIPPER, repeated incremental pruning to produce error reduction; MCC, Matthews correlation coefficient*.

### Results on BAF Dataset of CRC Stage

**Figure 2:**
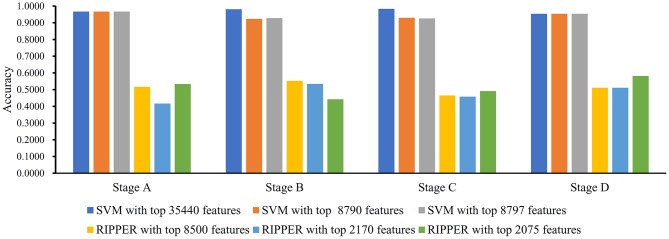
Bar chart to show accuracies on four CRC stages yielded by key support vector machine (SVM) and repeated incremental pruning to produce error reduction (RIPPER) classifiers on BAF data of CRC stage.

**Figure 3:**
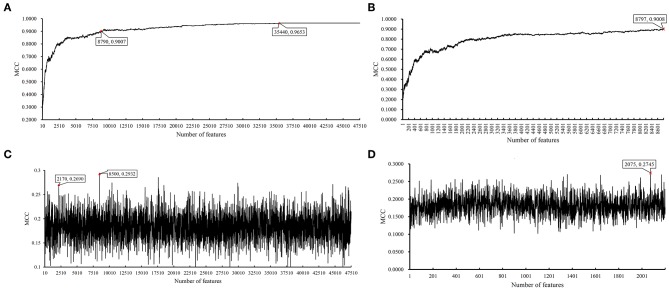
Incremental feature selection (IFS) results on BAF data of CRC stage yielded by SVM and RIPPER. **(A)** First-stage IFS results on BAF data of CRC stage yielded by SVM. **(B)** Second-stage IFS results on BAF data of CRC stage yielded by SVM. **(C)** First-stage IFS results on BAF data of CRC stage yielded by RIPPER. **(D)** Second-stage IFS results on BAF data of CRC stage yielded by RIPPER.

We first ran the computational pipeline on the first BAF dataset of CRC stage. Key results are provided in Table 1 and Figure 2. For the first stage of IFS with a step 10, results are provided in Table S5 and a curve with Matthews correlation coefficient (MCC) (Matthews, 1975; Gorodkin, 2004; Zhao et al., 2018, 2019; Cui and Chen, 2019) as Y-axis and number of features as X-axis was plot, as shown in Figure 3A. The SVM yielded the highest MCC value of 0.9653 (Table 1) when the top 35,440 features were used. Considering this extremely large number, we used another turning point (top 8,790 features), which still yielded a high MCC value of 0.9007. Thus, in the second IFS stage, we ran the same pipeline with the interval [1, 8800] with a step 1. Results are collected in Table S6, and a curve was also plotted, as shown in Figure 3B. The best MCC value was 0.9008 when the top 8,797 features were used. Accordingly, we built an optimal SVM classifier with the top 8,797 features.

In addition to SVM, we applied the interpretable rule learning method RIPPER to evaluate the selected features' performance in a rule manner. After running RIPPER on the samples consisting of features from individual feature subsets with a step 10, we obtained the performance of RIPPER on different feature subsets, as shown in Table S5 and Figure 3C. We obtained the best MCC value of 0.2932 when the top 8,500 features were used. A turning point was observed (top 2,170 features), yielding an MCC value of 0.2692. To further select the optimum features, we ran the IFS with RIPPER within the interval [1, 2,200]. Results are available in Table S6 and displayed in Figure 3D. We obtained the best MCC value of 0.2745 when the top 2,075 features were used.

Although RIPPER showed a poorer performance than SVM in this case, one advantage of RIPPER is that it can generate classification rules, which help us understand how the model makes a prediction on a subgroup of samples. Considering these data, the RIPPER produced 30 classification rules, which are given in Table S7.

### Results on LRR Dataset of CRC Stage

**Figure 4:**
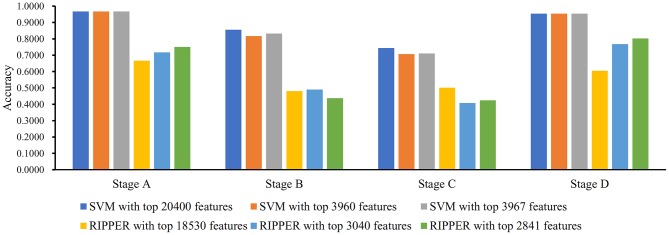
Bar chart to show accuracies on four CRC stages yielded by key SVM and RIPPER classifiers on LRR data of CRC stage.

**Figure 5:**
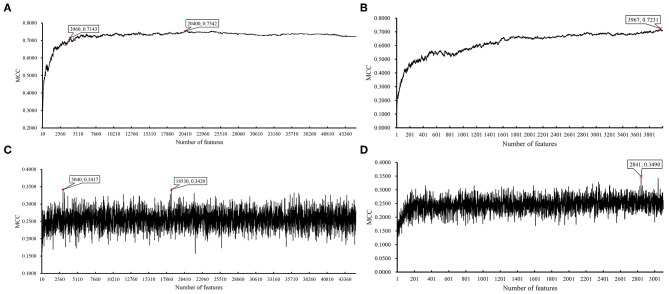
IFS results on LRR data of CRC stage yielded by SVM and RIPPER. **(A)** First-stage IFS results on LRR data of CRC stage yielded by SVM. **(B)** Second-stage IFS results on LRR data of CRC stage yielded by SVM. **(C)** First-stage IFS results on LRR data of CRC stage yielded by RIPPER. **(D)** Second-stage IFS results on LRR data of CRC stage yielded by RIPPER.

We ran the above same pipeline on the second dataset. Key results are provided in Table 2 and Figure 4. When running the IFS with an SVM on the samples consisting of features from individual feature subsets, we obtained the best MCC value of 0.7542 when the top 20,400 features were used. We adopted a smaller turning value (top 3,960 features), which yielded an MCC value of 0.7143. Then, we ran the second stage of IFS on the interval [1, 4000] and obtained the best MCC value of 0.7231 when the top 3,967 features were used. These results are given in Tables S8, S9 and illustrated in Figures 5A,B. Accordingly, an optimal SVM classifier was built based on the top 3,967 features.

**Table 2:** Performance of classification models on LRR dataset of CRC stage with IFS method.

**Classifier**	1st-stage IFS^*^		2nd-stage IFS^*^	**Number of rules**
	**Highest point**	**Turning point**		
SVM	0.7542 (20,400)	0.7143 (3,960)	0.7231 (3,967)	—
RIPPER	0.3420 (18,530)	0.3417 (3,040)	0.3490 (2,841)	32

* *These performances are measured by MCC; numbers of used features are listed in brackets*. *LRR, log R ratio*.

Similarly, IFS with RIPPER was also used on this dataset. All results are provided in Tables S8, S9 and displayed in Figures 5C,D. We obtained the best MCC value of 0.3420 when using the top 18,530 features. Of note, when 3,040 features were used, the performance showed a notable change as a performance turning point. Thus, in the second stage of IFS, we ran the RIPPER on the interval [1, 3100] and obtained the best MCC value of 0.3490 when using the top 2,841 features. The 32 learned classification rules are given in Table S10.

### Results on BAF Dataset of MACROD2 Status

**Figure 6:**
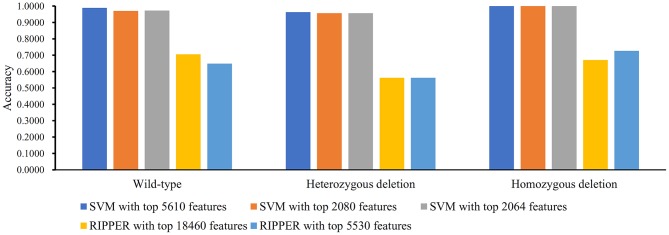
Bar chart to show accuracies on three MACROD2 status yielded by key SVM and RIPPER classifiers on BAF data of MACROD2 status.

**Figure 7:**
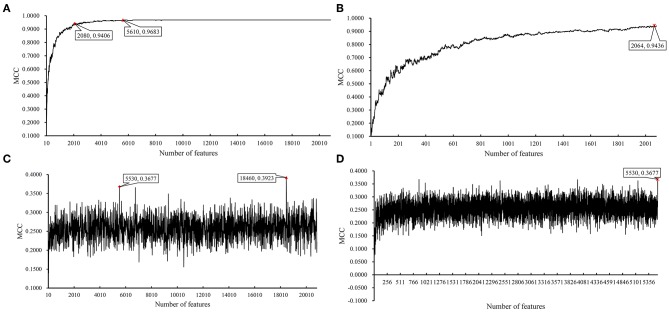
IFS results on BAF data of MACROD2 status yielded by SVM and RIPPER. **(A)** First-stage IFS results on BAF data of MACROD2 status yielded by SVM. **(B)** Second-stage IFS results on BAF data of MACROD2 status yielded by SVM. **(C)** First-stage IFS results on BAF data of MACROD2 status yielded by RIPPER. **(D)** Second-stage IFS results on BAF data of MACROD2 status yielded by RIPPER.

Instead of analyzing the association between the CRC stages and CNV states, we used the same pipeline to analyze the *MACROD2* status associated with particular CNV types. For the BAF dataset of *MACROD2* status, key results are provided in Table 3 and Figure 6. Results of the first stage of IFS with SVM are available in Table S11, and a curve was plotted in Figure 7A. We obtained the best MCC value of 0.9683 when the top 5,610 features were used. We detected the turning point 2,080, which yielded an MCC value of 0.9406. In the second stage of IFS, we ran the SVM on the interval [1, 2080]. Results are collected in Table S12, and a curve was plotted in Figure 7B. The best MCC value was 0.9436 when the top 2,064 features were used, which can be used to build an optimal SVM classifier.

**Table 3:** Performance of classification models on BAF dataset of MACROD2 status with IFS method.

**Classifier**	1st-stage IFS^*^		2nd-stage IFS^*^	**Number of rules**
	**Highest point**	**Turning point**		
SVM	0.9683 (5,610)	0.9406 (2,080)	0.9436 (2,064)	—
RIPPER	0.3923 (18,460)	0.3677 (5,530)	0.3677 (5,530)	23

* *These performances are measured by MCC; numbers of used features are listed in brackets*.

We also ran the IFS with RIPPER on this dataset. The first-stage results are provided in Table S11. A curve was plotted in Figure 7C. RIPPER yielded the best MCC value of 0.3923 when the top 18,460 features were used. We also selected the turning point 5530 for the second stage of IFS, which yielded an MCC value of 0.3677. For the second stage of IFS within the interval [1, 5530], results are available in Table S12 and a curve was shown in Figure 7D. We still obtained the best MCC value of 0.3677 when the top 5,530 features were used. The 23 classification rules generated by RIPPER are listed in Table S13.

### Results on LRR Dataset of MACROD2 Status

**Figure 8:**
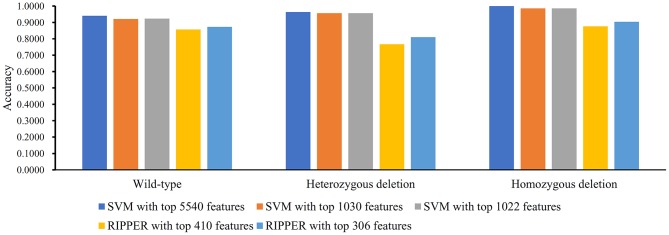
Bar chart to show accuracies on three MACROD2 status yielded by key SVM and RIPPER classifiers on LRR data of MACROD2 status.

**Figure 9:**
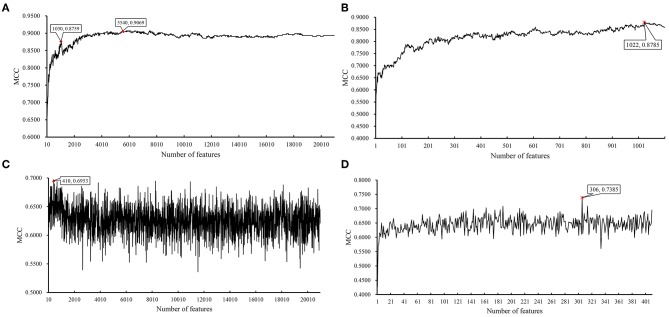
IFS results on LRR data of MACROD2 status yielded by SVM and RIPPER. **(A)** First-stage IFS results on LRR data of MACROD2 status yielded by SVM. **(B)** Second-stage IFS results on LRR data of MACROD2 status yielded by SVM. **(C)** First-stage IFS results on LRR data of MACROD2 status yielded by RIPPER. **(D)** Second-stage IFS results on LRR data of MACROD2 status yielded by RIPPER.

We did the similar procedures for the LRR dataset of *MACROD2* status. Key results are provided in Table 4 and Figure 8. For the first stage of IFS with SVM, results are provided in Table S14 and a curve was plotted in Figure 9A. We obtained the best MCC value of 0.9069 when using the top 5,540 features. Similarly, a smaller turning point 1,030 was used for the second stage of IFS, because it still yielded a satisfactory MCC value of 0.8759. In the second stage of IFS, we set the interval [1, 1,100]. Results are collected in Table S15, and a curve was plotted in Figure 9B. We obtained the best MCC value of 0.8785 when the top 1,022 features were adopted. The optimal SVM classifier was built using the top 1,022 features.

**Table 4:** Performance of classification models on LRR dataset of MACROD2 status with IFS method.

**Classifier**	1st-stage IFS^*^		2nd-stage IFS^*^	**Number of rules**
	**Highest point**	**Turning point**		
SVM	0.9069 (5,540)	0.8759 (1,030)	0.8785 (1,022)	—
RIPPER	0.6953 (410)	—	0.7385 (306)	17

* *These performances are measured by MCC; numbers of used features are listed in brackets*.

We ran the IFS with RIPPER again. Results are provided in Table S14. A curve was plotted in Figure 9C, from which we can see that the best MCC value was 0.6953 when the top 410 features were used. Then, we ran the second stage of IFS within the interval [1, 410]. Results are available in Table S15. A curve was plotted in Figure 9D. It can be seen that the best MCC value was 0.7385 when using the top 306 features. Table 5 lists the 17 classification rules generated by RIPPER.

**Table 5:** Classification rules on dataset of MACROD2 status with LRR.

**Index**	**Condition**	**Result**	**Support^**a**^%**	**Accuracy^**b**^%**
1	rs353149 <= −0.3811 rs6034087 <= −0.2724	Homozygous deletion	5.84	92.11
2	rs445945 <= −0.3040 rs6712905 >= 0.1367 rs377954 <= −0.3691	Homozygous deletion	3.23	90.48
3	rs6135314 <= −0.5109 rs6685801 <= −0.0057 rs2900712 <= −0.0619 rs9444675 <= 0.1020	Homozygous deletion	4.61	93.33
4	rs6135362 <= −0.2468 rs2100272 >= 0.1398 rs700029 <= 0.0035	Homozygous deletion	1.84	91.67
5	rs6110500 <= −0.2528 rs10500528 <= −0.0094	Homozygous deletion	7.37	83.33
6	rs6079537 <= −0.2319 rs2900712 <= −0.0981 rs6043173 >= −0.0832	Homozygous deletion	0.92	100.00
7	rs9355387 <= −0.2856 rs11905979 <= −0.3878	Homozygous deletion	1.84	91.67
8	rs199305 <= −0.4455 rs377201 >= −0.2189	Homozygous deletion	0.77	100.00
9	rs6135314 >= −0.0746 rs1998086 >= 0.0340 rs381053 >= −0.0576	Wild-type	35.48	98.70
10	rs1475531 >= −0.0454 rs365516 >= 0.0220	Wild-type	31.80	96.14
11	rs2423866 >= −0.1223 rs385770 >= −0.0670 rs7241111 >= −0.1500	Wild-type	24.42	98.11
12	rs449849 >= −0.0559 rs716316 >= −0.0107 rs5904713 >= −0.1428	Wild-type	27.80	97.24
13	rs1327323 <= −0.2719 rs6135269 <= −0.1044	Wild-type	6.76	75.00
14	rs353149 >= −0.0059 rs13011654 >= 0.0742 rs445945 <= 0.067	Wild-type	5.07	96.97
15	rs6034046 >= −0.015 rs6135314 >= −0.0323 rs6034011 <= 0.0668	Wild-type	19.05	95.16
16	rs6043173 >= 0.131 rs449849 >= −0.0689	Wild-type	23.81	94.84
17	Others	Heterozygous deletion	20.28	85.61

a *The support of a rule is the percentage of samples satisfying the rule*.

b *The accuracy of a rule is the proportion of the corrected classified samples among samples satisfying the rule*.

## Discussion

On each of four datasets, a group classification rules were generated by RIPPER. According to the performance of RIPPER listed in Table 4, rules on the LRR data of *MACROD2* status were with the highest performance (MCC = 0.7385). Thus, we mainly discussed these rules, which are listed in Table 5. Each rule can cover some CRC samples and give high accuracies.

Given that the status of *MACROD2* is significantly relevant to the intestinal tumorigenesis and plays a crucial role in cancer development (Sakthianandeswaren et al., 2018), our classifiers are expected to be prognostic indicators for evaluating the malignancy of intestinal tumor. On LRR data, 17 decision rules were generated by RIPPER, which can distinguish the three status of *MACROD2* with LRR with a classification accuracy of 0.7385. Depending on the CNV profiles of selected loci, predicting whether a heterozygous, or homozygous depletion of *MACROD2* exists in CRC patients is possible. To validate the reliability of these rules, we examined existing experimental evidence through a literature review.

We focused on the 17 decision rules and a few top-ranked features on data of *MACROD2* status with LRR. Such rules and features described specific CNV characteristics contributing to the identification of *MACROD2* status and CRC classification, indicating their crucial roles in cancer development. Especially, several top-ranked features showed strong biological and biomedical relevance with *MACROD2*, indicating that they also play relevant functions in cancer progression.

Among the 17 rules, 8 rules could identify the homozygous deletion of *MACROD2*, and the other 8 decision rules can identify the normal non-depletion status of *MACROD2*. The last one indicates the heterozygous deletion, which means that if the CNV profiles in patients failed to meet any criteria of the other 16 rules, they were predicted to carry the heterozygous deletion of *MACROD2*.

### Rules for Homozygous Deletion

In the eight rules identifying the homozygous deletion of *MACROD2* (see first eight rules in Table 5), 21 criteria involving 20 SNP sites were located in different regions of six genes. Notably, 12 of these SNP sites were located in the genomic regions of *MACROD2*, and the LRR of specific regions near these SNP sites featured a low value, which is naturally and logically consistent given that the CNV loss in *MACROD2* leads to homozygous deletion. Thus, our analysis actually highlights the potential core roles of specific SNP sites, suggesting its capability to identify the overall state of *MACROD2* based on the CNV conditions of a few loci. In detail, the 12 SNPs (rs353149, rs6034087, rs445945, rs377954, rs6135314, rs6135362, rs6110500, rs6079537, rs6043173, rs11905979, rs199305, and rs377201) were distributed in different locations of the intron regions of *MACROD2* and displayed strong relevance to the overall status of *MACROD2*. By the detection of CNV in these selected loci markers, we can identify the deletion state of *MACROD2* in patients. We will find the corresponding therapy methods for the treatment targets in the future. Further research about these incompletely elucidated SNP sites may reveal the mechanisms of tumor development at the genomic level. The biological and biomedical significance of several SNPs is summarized below.

The SNP site rs6685801 located in chr1:3547887 required a low value of LRR to identify the homozygous deletion of *MACROD2* in our decision rules. This position is in the intron region of multiple EGF-like-domains 6 (*MEGF6*) gene, which was reported to play a critical role in cell adhesion and involved in many disorders of neural system development (Sunnerhagen et al., 1993). Recent publications have confirmed that *MEFG6* can promote the epithelia-to-mesenchymal transition in CRC metastasis (Hu et al., 2018). This gene is also significantly upregulated in tumor tissue and results in the poor survival of a colon adenocarcinoma cohort. *MEGF6* can also accelerate the cell growth and inhibit apoptosis in CRC as demonstrated by the experiment *in vitro*. All these results suggest that *MEGF6* may serve as an oncogene, and its overexpression may contribute to the tumorigenesis in CRC patients. We inferred that the copy number loss in this specific intron region caused the upregulated expression of *MEGF6* as it may perform inhibitory effects on transcription. Thus, the low LRR of the SNP site rs6685801 can indicate the severe extent of CRC, consistent with the homozygous deletion state of *MACROD2*.

Another important SNP site rs9444675, which displayed strong relevance to the status of *MACROD2* in our classifier, is located in the intron region of gamma-aminobutyric acid receptor subunit rho-1 (*GABRR1*). *GABRR1*, also called GABA(A) receptor, is a member of the rho subunit family and acts as the receptor of major inhibitory neurotransmitters in the mammalian brain (Cutting et al., 1992). A recent study has shown that *GABRR1* is significantly upregulated by the transcriptome of chemokine (C-X-C motif) ligand 1-(*CXCL1*) treated colon cancer cells (Hsu et al., 2018). Further analysis via bioinformatics methods reported that high expression of *GABRR1* showed a significant correlation with reduced overall survival rates, suggesting the crucial role of *GABRR1* in the progression of colon cancer. In addition, another research reported the upregulation of *GABRR1* in cancer cohorts compared with the controls with regard to gene expression profiles of medullary thyroid carcinoma (Oczko-Wojciechowska et al., 2006). These pieces of evidences support the decision rule that copy number loss of specific region located in *GABRR1* will lead to the upregulation of *GABRR1* and contribute to the carcinogenesis of CRC, resulting in the similar consequence as the homozygous deletion state of *MACROD2*.

One important criterion identified in the decision rules suggests the high value of LRR near the specific SNP site rs2100272. This site is located in the intron regions of *VWA3B*, which showed a tendency toward malignancy development. *VWA3B* encodes an intracellular protein thought to function in transcription, DNA repair, and membrane transport (Kawarai et al., 2016; Huttlin et al., 2017), playing a role similar to *MACROD2*, which was reported to influence DNA repair and sensitivity to DNA damage and result in chromosome instability (Sakthianandeswaren et al., 2018). In the patients of bladder urothelial carcinoma, evident copy number alterations were observed in the 2q12 regions in which the *VWA3B* was mapped (E. Pontes et al., 2013), in line with the suggestion that VWA3B plays a crucial role in bladder carcinogenesis. In addition, *VWA3B* is significantly differentially expressed in tongue squamous cell carcinoma samples at the transcriptome level (Song et al., 2019). These results confirm our decision rules, which indicate that the copy number gain of the specific regions near rs2100272 will alter the expression of VWA3B and contribute to the development of certain cancers including CRC.

Another criterion was found in the experimental findings, and it required a low LRR near the SNP site rs700029 to identify the homozygous deletion state of *MACROD2*. This SNP site is located in chr1:81805339 and was mapped in the intron region of adhesion G protein-coupled receptor L2 (*ADGRL2*), which encodes a member of the latrophilin subfamily of G-protein coupled receptors. *ADGRL2* functions as a p53 target gene and regulator of neuronal exocytosis (Hamann et al., 2015). Recent research has shown the low expression level of ADGRL2 in genomic sequencing analyses of both gastric cancer and colon cancer cell lines due to the hypermethylation of CpG islands within the gene (Jeon et al., 2016). ADGRL2 is also associated with lung squamous cell carcinoma and may serve as the diagnostic marker for *small cell lung cancer* (Huang et al., 2018). The rules that require the copy number loss of specific intron region in ADGRL2 may result in the alteration of expression profile and lead to the development of CRC.

We also identified a critical SNP site rs9355387 located in the intron region of gene Parkin RBR E3 ubiquitin protein ligase (*PRKN*), which according to the rules indicates the homozygous deletion state of *MACROD2*. The gene *PRKN*, best known as *PARK2*, is a key component of a multiprotein E3 ubiquitin ligase complex, which mediates the targeting of substrate proteins for proteasomal degradation. Mutations occurring in this gene cause Parkinson's disease (Oczkowska et al., 2013). The loss of *PRKN* at both the DNA copy number and mRNA expression levels contributes to cancer progression via redox-mediated inactivation of phosphatase and tensin homolog (*PTEN*) (Gupta et al., 2017). The depletion of *PRKN* also enhanced pancreatic tumorigenesis in KRAS-driven engineered mouse models based on its role in mediating the degradation of mitochondrial iron importers (Kang et al., 2019), implying that *PRKN* can be a potential target for pancreatic cancer therapy. These results highlight the crucial role of *PRKN* in cancer progression and confirm our predicted rules, indicating that the loss of copy number near rs9355387 would be an indicator of severe status of cancer.

### Rules for Wild-Type

The eight rules for identifying the non-deletion or wild-type status of *MACROD2* included 21 criteria with 19 SNP sites, 15 of which are located in the intron regions of *MACROD2*. The LRR of these specific regions requires a high value opposite that of the homozygous deletion state. Among the 15 SNP sites located in *MACROD2* and with built non-deletion status, 4 SNPs (rs6135314, rs353149, rs445945, and rs6043173) have been applied in the identification of the homozygous deletion state of *MACROD2* with relatively low values as mentioned before. The other 11 SNP sites (rs1998086, rs381053, rs1475531, rs365516, rs2423866, rs385770, rs449849, rs716316, rs6135269, rs6034046, and rs6034011) showed different distributions in varying locations in the intron regions of *MACROD2*, displaying a significant correlation with the overall state of *MACROD2* and implying that these selected loci may play unexplained functional roles in regulating DNA replication. The candidate SNP sites identified by our prediction model can be applied as biomarkers for the pathologic evaluation of CRC, given that the state of *MACROD2* has been confirmed to be a significant signal in intestinal cancers.

The copy number loss of the regions near the SNP site rs1327323 can indicate the non-deletion state of *MACROD2* in one decision rule. This site is located in chr13:52296316 and mapped in the intron regions of transmembrane phosphoinositide 3-phosphatase and tensin homolog 2 pseudogene 2 (*TPTE2P2*), which is considered a putative promoter in human genome (Kimura et al., 2006). By the whole-exome sequencing analysis of 42 tumor–normal paired samples, highly frequent sites of increased copy number were found in the specific position of chromosome arm 13q (Corraliza Márquez, 2014), the gains in which have been associated with a poor prognosis and metastasis in CRC (Leary et al., 2008). *TPTE2P2* is present in the segments with copy number loss, suggesting that it probably facilitates defect in tumorigenesis. Another publication also reported *TPTE2P2* as one of the key genes identified in gastric cancers (Zeng et al., 2018), implying its crucial role in certain cancers. We inferred that the copy number gain in the specific intron region of *TPTE2P2* results in the progression of CRC, and the loss of copy number in our decision rules identifies the normal status of *MACROD2* and the absence of CRC.

Some SNP sites (rs5904713 and rs13011654) are located in the intron regions of the non-coding RNA gene or the intergenic regions in our decision rules. They have not been reported in current research literature but show strong relevance to the progression of CRC at the CNV level, implying their potential roles in the regulation of oncogenes.

Numerous top-ranked features display the significant relevance to the classification of three status of *MACROD2*, most of which are located in the intron regions of *MACROD2*. Coincident with the relevant information and our inferred decision rules, the CNVs in *MACROD2* resulted in the direct altered states (e.g., cancer). In addition, our approach provides an effective method to evaluate the malignancy extent by detecting a few biomarkers (e.g., SNP sites) rather than conducting an overall detailed analysis of the large gene *MACROD2*, which is more than two million base pairs in size. In summary, our study has proposed for the first time that specific SNP sites can be applied as biomarkers in cancer diagnosis, and further research on these sites will shed light on the molecular mechanism on how these specific DNA regions contribute to the progression of CRC.

## Data Availability Statement

Publicly available datasets were analyzed in this study. This data can be found here: https://www.ncbi.nlm.nih.gov/geo/query/acc.cgi?acc=GSE115145.

## Author Contributions

TH and Y-DC designed the study. SZ, XP, and LC performed the experiments. TZ, WG, ZG, Y-HZ, and YZ analyzed the results. SZ, XP, and TZ wrote the manuscript. All authors contributed to the research and reviewed the manuscript.

### Conflict of Interest

The authors declare that the research was conducted in the absence of any commercial or financial relationships that could be construed as a potential conflict of interest.

## Supplementary Material

Table S1 Ranked features with MaxRel scores for CRC stage with BAF.

Table S2 Ranked features with MaxRel scores for CRC stage with LRR.

Table S3 Ranked features with MaxRel scores for MACROD2 status with BAF.

Table S4 Ranked features with MaxRel scores for MACROD2 status with LRR.

Table S5 Performance of 1st-stage IFS with SVM and RIPPER for CRC stage with BAF.

Table S6 Performance of 2nd-stage IFS with SVM and RIPPER for CRC stage with BAF.

Table S7 Classification rules learned by RIPPER for CRC stage with BAF.

Table S8 Performance of 1st-stage IFS with SVM and RIPPER for CRC stage with LRR.

Table S9 Performance of 2nd-stage IFS with SVM and RIPPER for CRC stage with LRR.

Table S10 Classification rules learned by RIPPER for CRC stage with LRR.

Table S11 Performance of 1st-stage IFS with SVM and RIPPER for MACROD2 status with BAF.

Table S12 Performance of 2nd-stage IFS with SVM and RIPPER for MACROD2 status with BAF.

Table S13 Classification rules learned by RIPPER for MACROD2 status with BAF.

Table S14 Performance of 1st-stage IFS with SVM and RIPPER for MACROD2 status with LRR.

Table S15 Performance of 2nd-stage IFS with SVM and RIPPER for MACROD2 status with LRR.
